# Methyl (3*S*,3′*R*)-1-methyl-2,2′′-dioxo-1′,2′,3′,5′,6′,7′,8′,8a′-octa­hydro­dispiro­[indoline-3,2′-indolizine-3′,3′′-indoline]-1′-carboxyl­ate

**DOI:** 10.1107/S1600536812037531

**Published:** 2012-09-08

**Authors:** G. Ganesh, Panneer Selvam Yuvaraj, E. Govindan, Boreddy S. R. Reddy, A. SubbiahPandi

**Affiliations:** a Department of Physics, S.M.K. Fomra Institute of Technology, Thaiyur, Chennai 603 103, India; b Industrial Chemistry Laboratory, Central Leather Research Institute, Adyar, Chennai 600 020, India; c Department of Physics, Presidency College (Autonomous), Chennai 600 005, India

## Abstract

In the title compound, C_25_H_25_N_3_O_4_, the central pyrrolidine ring and the two pyrrolidine rings adopt twisted conformations, whereas the piperidine ring in the octa­hydro­indolizine fused ring system adopts a chair conformation. The indoline ring systems are almost perpendicular with respect to the mean plane of the octa­hydro­indolizine ring system, making dihedral angles of 84.4 (5) and 79.4 (5)°. The acetate group attached to the octa­hydro­indolizine ring system assumes an extended conformation. In the crystal, N—H⋯O hydrogen bonds result in the formation of a helical *C*(7) chain running parallel to [101]. The crystal packing features C—H⋯O hydrogen bonds and C—H⋯π inter­actions.

## Related literature

For the biological activity of compounds with spiro-pyrrolidine ring systems, see: Sundar *et al.* (2011 ▶); Crooks & Sommerville (1982 ▶); Stylianakis *et al.* (2003 ▶). For a related structure, see: Selvanayagam *et al.* (2012 ▶). For puckering parameters, see: Cremer & Pople (1975 ▶). For asymmetry parameters, see: Nardelli *et al.* (1983 ▶).
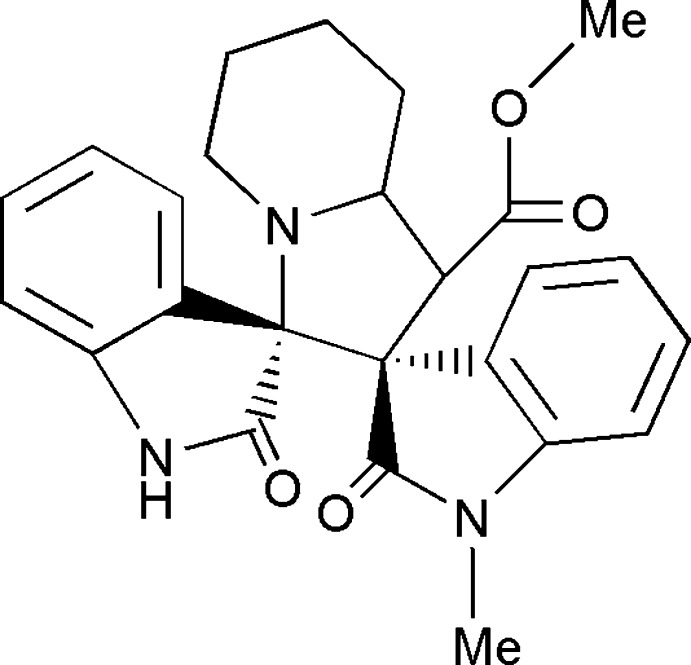


## Experimental

### 

#### Crystal data

C_25_H_25_N_3_O_4_
*M* *_r_* = 431.48
Monoclinic, 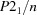

*a* = 10.0516 (3) Å
*b* = 17.9539 (6) Å
*c* = 12.4471 (4) Å
β = 105.347 (2)°
*V* = 2166.17 (12) Å^3^
*Z* = 4
Mo *K*α radiation
μ = 0.09 mm^−1^
*T* = 293 K
0.25 × 0.22 × 0.19 mm

#### Data collection

Bruker APEXII CCD area-detector diffractometer
Absorption correction: multi-scan (*SADABS*; Sheldrick, 1996 ▶) *T*_min_ = 0.978, *T*_max_ = 0.983
27282 measured reflections
6752 independent reflections
4374 reflections with *I* > 2σ(*I*)
*R*_int_ = 0.033

#### Refinement

*R*[*F* ^2^ > 2σ(*F* ^2^)] = 0.049
*wR*(*F* ^2^) = 0.143
*S* = 1.03
6752 reflections
291 parameters
H-atom parameters constrained
Δρ_max_ = 0.23 e Å^−3^
Δρ_min_ = −0.24 e Å^−3^

### 

Data collection: *APEX2* (Bruker, 2008 ▶); cell refinement: *SAINT* (Bruker, 2008 ▶); data reduction: *SAINT*; program(s) used to solve structure: *SHELXS97* (Sheldrick, 2008 ▶); program(s) used to refine structure: *SHELXL97* (Sheldrick, 2008 ▶); molecular graphics: *ORTEP-3* (Farrugia, 1997 ▶); software used to prepare material for publication: *SHELXL97* and *PLATON* (Spek, 2009 ▶).

## Supplementary Material

Crystal structure: contains datablock(s) global, I. DOI: 10.1107/S1600536812037531/zl2499sup1.cif

Structure factors: contains datablock(s) I. DOI: 10.1107/S1600536812037531/zl2499Isup2.hkl

Additional supplementary materials:  crystallographic information; 3D view; checkCIF report

## Figures and Tables

**Table 1:** Hydrogen-bond geometry (Å, °) *Cg*6 is the centroid of the C18–C23 ring.

*D*—H⋯*A*	*D*—H	H⋯*A*	*D*⋯*A*	*D*—H⋯*A*
N2—H2*C*⋯O4^i^	0.86	2.21	2.9639 (15)	146
C2—H2*A*⋯O3^ii^	0.97	2.48	3.348 (2)	150
C25—H25*C*⋯*Cg*6^iii^	0.96	2.81	3.5617 (19)	135

Symmetry codes: (i) 
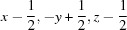
; (ii) 
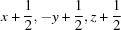
; (iii) 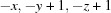.

**Fig. 1.**
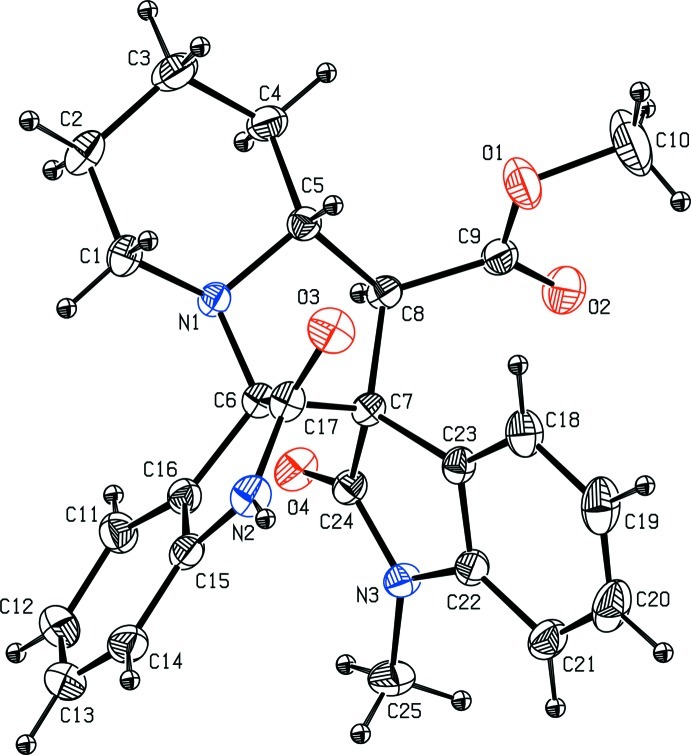
The structure of the title compound showing the atom-numbering scheme. The displacement ellipsoids are drawn at the 30% probability level.

**Fig. 2.**
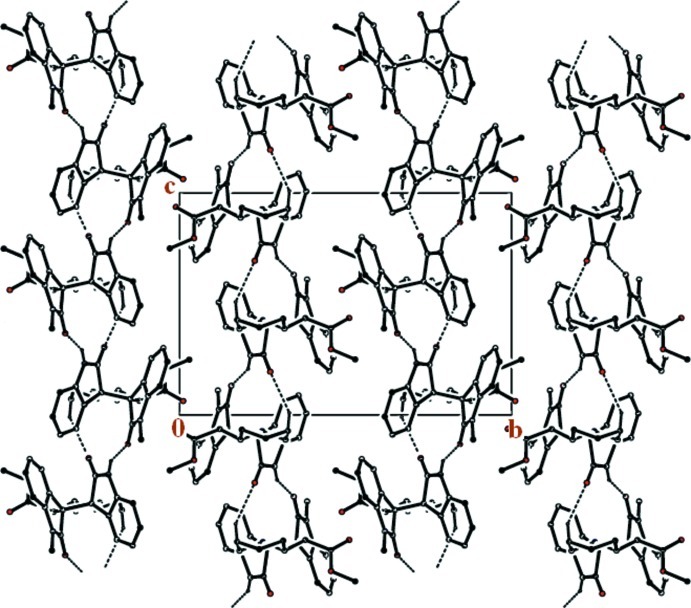
The molecular packing in which the direction of the chains along the a-c diagonal axis is shown. Dashed lines show the intermolecular C—H···O and N—H···O hydrogen bonds.

## supplementary crystallographic information

### Comment

The spiro-pyrrolidine ring system is a structural motif present in many biologically important and pharmacologically relevant alkaloids. Some derivatives are used as antimicrobial and antitumour agents (Sundar *et al.*, 2011), or possess analgesic (Crooks & Sommerville, 1982) and anti-influenza virus (Stylianakis *et al.*, 2003) activities. In view of this importance and in continuation of our work on the crystal structure analyis of spiro-pyrrolidine derivatives, the crystal structure of the title compound has been determined and the results are presented here.

X-Ray analysis confirms the molecular structure and atom connectivity as illustrated in Fig. 1. The geometry of the pyrrolidine and indoline group systems are comparable with those of related structures (Selvanayagam *et al.*, 2012). The sum of the angles at N1 [336.3 (1)°], N2[360.0 (1)°] and N3 [359.8 (1)°] of the pyrrolidine rings are in accordance with *sp*^3^ hybridizations for N1 and *sp*^2^ hybridizations for N2 and N3. The indoline ring systems [N2/C6/C11—C17 and N3/C7/C18—C24] make dihedral angles of 84.4 (5)° and 79.4 (5)° with respect to the mean plane of the octahydroindolizine ring system, which clearly shows the indoline rings attached to the octahydroindolizine ring system are almost perpendicular to each other. The acetate group assumes an extended conformation as can be seen from the torsion angle C8—C9—O1—C10 = -177.9 (2)°.

The pyrrolidine rings [N1/C5—C8, N2/C6/C15—C17 and N3/C7/C22—C24] adopt twisted conformations, with puckering parameters q_2_ and φ (Cremer & Pople, 1975) and the smallest displacement asymmetric parameters, Δ, (Nardelli *et al.*, 1983) as follows: q_2_ = 0.4349 (1) Å; 0.1063 (1) Å & 0.1221 (1) Å, φ = 194.8 (2)°; 123.4 (8)° & 127.6 (7)°, Δ_2_[(C7) = 4.86 (12)°]; [(C15) = 0.51 (15)] & [(C22) = 1.72 (14)]. The piperidine ring adopts a chair conformation, with the puckering parameters q_2_ = 0.0284 (2) Å; q_3_ = -0.5721 (2) Å & φ = 118 (3) ° and the smallest displacement asymmetric parameter, Δ_s_ (C2 & C5) = 2.32 (13)°.

Two intermolecular N2—H2C···O4 (-1/2 + *x*,1/2 - *y*,-1/2 + *z*) and C2—H2A···O3 (1/2 + *x*,1/2 - *y*,1/2 + *z*) hydrogen bonds both result in the formation of helicsu2496al C^1^_1_(7) chains running running parallel to [1 0 1]. The crystal packing is stabilized by C—H···O, N—H···O and C–H···π interactions (Table. 1).

### Experimental

A mixture of 1eq of (*E*)-methyl 2-(1-methyl-2-oxoindolin-3-ylidene) acetate, 1eq of isatin and 1.5eq of pipecolinic acid were dissolved in acetonitrile. This reaction mixture was refluxed at 80°C for 8 h. Completion of the reaction was monitored by thin layer chromatography. The reaction mixture was taken up in water, extracted with ethyl acetate and washed with water. The product was dried and purified by column chromatography using ethyl acetate and hexanes (1:9) as the elutent to afford pure dispiro oxindole (yield: 78%, M.p.: 541 K). Single crystals suitable for X-ray diffraction were obtained by slow evaporation of a solution of the title compound in ethyl acetate at room temperature.

### Refinement

All H atoms were fixed geometrically and allowed to ride on their parent atoms, with C—H distances fixed in the range 0.93–0.97 Å and N—H distances of 0.86 ° with *U*_iso_(H) = 1.5*U*_eq_(C) for methyl H and 1.2*U*_eq_(C/N) for all other H atoms.

### Crystal data

**Table:**

C_25_H_25_N_3_O_4_	*F*(000) = 912
*M**_r_* = 431.48	*D*_x_ = 1.323 Mg m^−^^3^
Monoclinic, *P*2_1_/*n*	Mo *K*α radiation, λ = 0.71073 Å
Hall symbol: -P 2yn	Cell parameters from 6752 reflections
*a* = 10.0516 (3) Å	θ = 2.0–31.0°
*b* = 17.9539 (6) Å	µ = 0.09 mm^−^^1^
*c* = 12.4471 (4) Å	*T* = 293 K
β = 105.347 (2)°	Block, white crystalline
*V* = 2166.17 (12) Å^3^	0.25 × 0.22 × 0.19 mm
*Z* = 4	

### Data collection

**Table:**

Bruker APEXII CCD area-detector diffractometer	6752 independent reflections
Radiation source: fine-focus sealed tube	4374 reflections with *I* > 2σ(*I*)
Graphite monochromator	*R*_int_ = 0.033
ω and φ scans	θ_max_ = 31.0°, θ_min_ = 2.0°
Absorption correction: multi-scan (*SADABS*; Sheldrick, 1996)	*h* = −14→14
*T*_min_ = 0.978, *T*_max_ = 0.983	*k* = −25→25
27282 measured reflections	*l* = −17→17

### Refinement

**Table:**

Refinement on *F*^2^	Primary atom site location: structure-invariant direct methods
Least-squares matrix: full	Secondary atom site location: difference Fourier map
*R*[*F*^2^ > 2σ(*F*^2^)] = 0.049	Hydrogen site location: inferred from neighbouring sites
*wR*(*F*^2^) = 0.143	H-atom parameters constrained
*S* = 1.03	*w* = 1/[σ^2^(*F*_o_^2^) + (0.0675*P*)^2^ + 0.2828*P*] where *P* = (*F*_o_^2^ + 2*F*_c_^2^)/3
6752 reflections	(Δ/σ)_max_ < 0.001
291 parameters	Δρ_max_ = 0.23 e Å^−^^3^
0 restraints	Δρ_min_ = −0.24 e Å^−^^3^

### Special details

**Table:**

Geometry. All e.s.d.'s (except the e.s.d. in the dihedral angle between two l.s. planes) are estimated using the full covariance matrix. The cell e.s.d.'s are taken into account individually in the estimation of e.s.d.'s in distances, angles and torsion angles; correlations between e.s.d.'s in cell parameters are only used when they are defined by crystal symmetry. An approximate (isotropic) treatment of cell e.s.d.'s is used for estimating e.s.d.'s involving l.s. planes.
Refinement. Refinement of *F*^2^ against ALL reflections. The weighted *R*-factor *wR* and goodness of fit *S* are based on *F*^2^, conventional *R*-factors *R* are based on *F*, with *F* set to zero for negative *F*^2^. The threshold expression of *F*^2^ > σ(*F*^2^) is used only for calculating *R*-factors(gt) *etc*. and is not relevant to the choice of reflections for refinement. *R*-factors based on *F*^2^ are statistically about twice as large as those based on *F*, and *R*- factors based on ALL data will be even larger.

### Fractional atomic coordinates and isotropic or equivalent isotropic displacement parameters (Å^2^)

**Table:**

	*x*	*y*	*z*	*U*_iso_*/*U*_eq_	
C1	0.39509 (15)	0.18055 (7)	0.38847 (13)	0.0389 (3)	
H1A	0.3661	0.1786	0.3077	0.047*	
H1B	0.3520	0.1394	0.4172	0.047*	
C2	0.55128 (16)	0.17364 (9)	0.42873 (14)	0.0475 (4)	
H2A	0.5783	0.1691	0.5092	0.057*	
H2B	0.5803	0.1288	0.3979	0.057*	
C3	0.62352 (16)	0.24026 (9)	0.39500 (16)	0.0528 (4)	
H3A	0.6072	0.2411	0.3146	0.063*	
H3B	0.7222	0.2362	0.4279	0.063*	
C4	0.57047 (14)	0.31205 (8)	0.43361 (14)	0.0421 (3)	
H4A	0.5954	0.3137	0.5144	0.051*	
H4B	0.6125	0.3545	0.4071	0.051*	
C5	0.41569 (13)	0.31611 (7)	0.38920 (11)	0.0312 (3)	
H5	0.3898	0.3183	0.3076	0.037*	
C6	0.20445 (12)	0.26486 (7)	0.39870 (10)	0.0296 (3)	
C7	0.19738 (12)	0.35157 (7)	0.42780 (10)	0.0287 (3)	
C8	0.34676 (13)	0.37893 (7)	0.43616 (11)	0.0314 (3)	
H8	0.3936	0.3815	0.5159	0.038*	
C9	0.35351 (14)	0.45585 (7)	0.39114 (14)	0.0411 (3)	
C11	0.14592 (16)	0.18861 (8)	0.56175 (13)	0.0434 (3)	
H11	0.2242	0.2033	0.6161	0.052*	
C12	0.04945 (18)	0.14130 (9)	0.58827 (16)	0.0545 (4)	
H12	0.0624	0.1252	0.6613	0.065*	
C13	−0.06500 (17)	0.11821 (9)	0.50720 (17)	0.0571 (5)	
H13	−0.1290	0.0872	0.5268	0.068*	
C14	−0.08675 (15)	0.14005 (8)	0.39767 (15)	0.0475 (4)	
H14	−0.1628	0.1233	0.3428	0.057*	
C15	0.00836 (13)	0.18761 (7)	0.37260 (12)	0.0357 (3)	
C16	0.12326 (13)	0.21318 (7)	0.45356 (11)	0.0332 (3)	
C17	0.12949 (14)	0.25295 (7)	0.27335 (11)	0.0349 (3)	
C18	0.04727 (17)	0.41569 (8)	0.24454 (12)	0.0447 (3)	
H18	0.1102	0.4081	0.2027	0.054*	
C19	−0.07771 (19)	0.45125 (9)	0.19857 (15)	0.0575 (5)	
H19	−0.0988	0.4670	0.1248	0.069*	
C20	−0.17011 (18)	0.46340 (10)	0.25991 (16)	0.0591 (5)	
H20	−0.2529	0.4873	0.2269	0.071*	
C21	−0.14357 (15)	0.44103 (8)	0.36957 (15)	0.0485 (4)	
H21	−0.2065	0.4495	0.4112	0.058*	
C22	−0.01959 (13)	0.40548 (7)	0.41489 (12)	0.0344 (3)	
C23	0.07557 (13)	0.39205 (7)	0.35351 (11)	0.0325 (3)	
C24	0.16580 (13)	0.35799 (7)	0.54149 (11)	0.0315 (3)	
N1	0.35207 (10)	0.25114 (6)	0.42717 (9)	0.0302 (2)	
N2	0.01226 (12)	0.21398 (7)	0.26806 (10)	0.0399 (3)	
H2C	−0.0517	0.2065	0.2077	0.048*	
N3	0.03374 (11)	0.38133 (6)	0.52466 (10)	0.0349 (3)	
O1	0.38982 (14)	0.45660 (6)	0.29638 (11)	0.0609 (3)	
O2	0.33032 (14)	0.51090 (6)	0.43692 (13)	0.0672 (4)	
O3	0.16969 (12)	0.27330 (6)	0.19483 (8)	0.0488 (3)	
O4	0.24468 (10)	0.34408 (6)	0.63171 (8)	0.0432 (2)	
C25	−0.03937 (16)	0.38432 (10)	0.61061 (14)	0.0509 (4)	
H25A	0.0221	0.3706	0.6809	0.076*	
H25B	−0.1159	0.3504	0.5924	0.076*	
H25C	−0.0727	0.4340	0.6154	0.076*	
C10	0.4028 (3)	0.52914 (11)	0.2504 (2)	0.0971 (8)	
H10A	0.3167	0.5553	0.2378	0.146*	
H10B	0.4268	0.5236	0.1811	0.146*	
H10C	0.4737	0.5569	0.3017	0.146*	

### Atomic displacement parameters (Å^2^)

**Table:**

	*U*^11^	*U*^22^	*U*^33^	*U*^12^	*U*^13^	*U*^23^
C1	0.0442 (8)	0.0309 (6)	0.0414 (8)	0.0046 (6)	0.0107 (6)	−0.0048 (6)
C2	0.0450 (8)	0.0427 (8)	0.0523 (9)	0.0141 (6)	0.0088 (7)	−0.0067 (7)
C3	0.0377 (8)	0.0529 (9)	0.0708 (11)	0.0085 (7)	0.0194 (8)	−0.0090 (8)
C4	0.0315 (7)	0.0413 (7)	0.0546 (9)	0.0007 (6)	0.0134 (6)	−0.0045 (7)
C5	0.0314 (6)	0.0300 (6)	0.0329 (7)	0.0007 (5)	0.0099 (5)	−0.0019 (5)
C6	0.0296 (6)	0.0300 (6)	0.0278 (6)	−0.0002 (5)	0.0050 (5)	−0.0023 (5)
C7	0.0267 (6)	0.0308 (6)	0.0269 (6)	0.0012 (5)	0.0042 (5)	−0.0024 (5)
C8	0.0281 (6)	0.0311 (6)	0.0345 (7)	−0.0006 (5)	0.0072 (5)	−0.0050 (5)
C9	0.0332 (7)	0.0304 (7)	0.0605 (10)	−0.0013 (5)	0.0137 (7)	−0.0040 (6)
C11	0.0428 (8)	0.0429 (8)	0.0431 (8)	−0.0014 (6)	0.0086 (6)	0.0068 (6)
C12	0.0558 (10)	0.0529 (9)	0.0585 (11)	0.0014 (8)	0.0218 (8)	0.0191 (8)
C13	0.0422 (9)	0.0482 (9)	0.0847 (14)	−0.0038 (7)	0.0235 (9)	0.0130 (9)
C14	0.0323 (7)	0.0394 (8)	0.0696 (11)	−0.0032 (6)	0.0114 (7)	−0.0023 (7)
C15	0.0296 (6)	0.0304 (6)	0.0457 (8)	0.0032 (5)	0.0073 (6)	−0.0040 (6)
C16	0.0320 (6)	0.0295 (6)	0.0371 (7)	−0.0001 (5)	0.0077 (5)	−0.0007 (5)
C17	0.0362 (7)	0.0333 (6)	0.0312 (7)	0.0025 (5)	0.0021 (5)	−0.0042 (5)
C18	0.0539 (9)	0.0390 (8)	0.0361 (8)	0.0001 (6)	0.0031 (7)	0.0017 (6)
C19	0.0641 (11)	0.0469 (9)	0.0455 (9)	0.0034 (8)	−0.0136 (8)	0.0082 (7)
C20	0.0417 (9)	0.0468 (9)	0.0727 (13)	0.0083 (7)	−0.0129 (8)	0.0059 (8)
C21	0.0304 (7)	0.0407 (8)	0.0698 (11)	0.0028 (6)	0.0053 (7)	0.0004 (8)
C22	0.0288 (6)	0.0293 (6)	0.0423 (8)	−0.0009 (5)	0.0046 (6)	−0.0011 (5)
C23	0.0308 (6)	0.0299 (6)	0.0333 (7)	0.0006 (5)	0.0020 (5)	−0.0005 (5)
C24	0.0306 (6)	0.0311 (6)	0.0319 (7)	0.0016 (5)	0.0067 (5)	−0.0028 (5)
N1	0.0283 (5)	0.0279 (5)	0.0333 (6)	0.0015 (4)	0.0061 (4)	−0.0021 (4)
N2	0.0341 (6)	0.0414 (6)	0.0375 (6)	−0.0029 (5)	−0.0020 (5)	−0.0079 (5)
N3	0.0319 (6)	0.0375 (6)	0.0366 (6)	0.0026 (5)	0.0115 (5)	−0.0006 (5)
O1	0.0904 (9)	0.0347 (6)	0.0664 (8)	−0.0031 (6)	0.0362 (7)	0.0076 (5)
O2	0.0742 (8)	0.0343 (6)	0.1035 (11)	0.0019 (5)	0.0418 (8)	−0.0131 (6)
O3	0.0554 (6)	0.0600 (7)	0.0290 (5)	−0.0038 (5)	0.0078 (5)	−0.0021 (5)
O4	0.0426 (6)	0.0548 (6)	0.0282 (5)	0.0092 (5)	0.0024 (4)	−0.0019 (4)
C25	0.0466 (9)	0.0592 (10)	0.0551 (10)	0.0026 (7)	0.0280 (8)	−0.0015 (8)
C10	0.149 (2)	0.0460 (11)	0.110 (2)	−0.0063 (13)	0.0586 (18)	0.0246 (12)

### Geometric parameters (Å, º)

**Table:**

C1—N1	1.4613 (16)	C12—H12	0.9300
C1—C2	1.521 (2)	C13—C14	1.379 (3)
C1—H1A	0.9700	C13—H13	0.9300
C1—H1B	0.9700	C14—C15	1.3778 (19)
C2—C3	1.515 (2)	C14—H14	0.9300
C2—H2A	0.9700	C15—C16	1.394 (2)
C2—H2B	0.9700	C15—N2	1.3949 (19)
C3—C4	1.521 (2)	C17—O3	1.2087 (17)
C3—H3A	0.9700	C17—N2	1.3570 (18)
C3—H3B	0.9700	C18—C23	1.3772 (19)
C4—C5	1.5087 (19)	C18—C19	1.390 (2)
C4—H4A	0.9700	C18—H18	0.9300
C4—H4B	0.9700	C19—C20	1.367 (3)
C5—N1	1.4672 (15)	C19—H19	0.9300
C5—C8	1.5193 (16)	C20—C21	1.380 (3)
C5—H5	0.9800	C20—H20	0.9300
C6—N1	1.4525 (15)	C21—C22	1.3807 (19)
C6—C16	1.5126 (17)	C21—H21	0.9300
C6—C17	1.5574 (19)	C22—C23	1.3942 (18)
C6—C7	1.6041 (17)	C22—N3	1.3980 (18)
C7—C23	1.5109 (18)	C24—O4	1.2169 (16)
C7—C24	1.5344 (17)	C24—N3	1.3543 (16)
C7—C8	1.5569 (17)	N2—H2C	0.8600
C8—C9	1.4987 (18)	N3—C25	1.4503 (17)
C8—H8	0.9800	O1—C10	1.442 (2)
C9—O2	1.1942 (17)	C25—H25A	0.9600
C9—O1	1.3237 (19)	C25—H25B	0.9600
C11—C16	1.378 (2)	C25—H25C	0.9600
C11—C12	1.393 (2)	C10—H10A	0.9600
C11—H11	0.9300	C10—H10B	0.9600
C12—C13	1.378 (3)	C10—H10C	0.9600
			
N1—C1—C2	109.38 (11)	C12—C13—H13	119.3
N1—C1—H1A	109.8	C14—C13—H13	119.3
C2—C1—H1A	109.8	C15—C14—C13	117.52 (15)
N1—C1—H1B	109.8	C15—C14—H14	121.2
C2—C1—H1B	109.8	C13—C14—H14	121.2
H1A—C1—H1B	108.2	C14—C15—C16	122.15 (14)
C3—C2—C1	111.89 (13)	C14—C15—N2	127.88 (14)
C3—C2—H2A	109.2	C16—C15—N2	109.88 (12)
C1—C2—H2A	109.2	C11—C16—C15	119.47 (13)
C3—C2—H2B	109.2	C11—C16—C6	131.94 (12)
C1—C2—H2B	109.2	C15—C16—C6	108.57 (12)
H2A—C2—H2B	107.9	O3—C17—N2	126.05 (13)
C2—C3—C4	110.35 (12)	O3—C17—C6	126.30 (12)
C2—C3—H3A	109.6	N2—C17—C6	107.64 (11)
C4—C3—H3A	109.6	C23—C18—C19	118.39 (15)
C2—C3—H3B	109.6	C23—C18—H18	120.8
C4—C3—H3B	109.6	C19—C18—H18	120.8
H3A—C3—H3B	108.1	C20—C19—C18	121.13 (16)
C5—C4—C3	109.82 (12)	C20—C19—H19	119.4
C5—C4—H4A	109.7	C18—C19—H19	119.4
C3—C4—H4A	109.7	C19—C20—C21	121.61 (15)
C5—C4—H4B	109.7	C19—C20—H20	119.2
C3—C4—H4B	109.7	C21—C20—H20	119.2
H4A—C4—H4B	108.2	C20—C21—C22	117.14 (15)
N1—C5—C4	109.79 (11)	C20—C21—H21	121.4
N1—C5—C8	100.62 (9)	C22—C21—H21	121.4
C4—C5—C8	115.22 (11)	C21—C22—C23	122.14 (14)
N1—C5—H5	110.3	C21—C22—N3	127.87 (13)
C4—C5—H5	110.3	C23—C22—N3	109.91 (11)
C8—C5—H5	110.3	C18—C23—C22	119.57 (13)
N1—C6—C16	115.10 (10)	C18—C23—C7	132.35 (12)
N1—C6—C17	114.40 (10)	C22—C23—C7	108.08 (11)
C16—C6—C17	101.08 (10)	O4—C24—N3	125.36 (12)
N1—C6—C7	102.30 (9)	O4—C24—C7	126.32 (11)
C16—C6—C7	115.59 (10)	N3—C24—C7	108.31 (11)
C17—C6—C7	108.71 (10)	C6—N1—C1	116.04 (10)
C23—C7—C24	101.32 (10)	C6—N1—C5	106.90 (10)
C23—C7—C8	120.00 (11)	C1—N1—C5	113.03 (10)
C24—C7—C8	110.24 (10)	C17—N2—C15	111.60 (11)
C23—C7—C6	113.94 (10)	C17—N2—H2C	124.2
C24—C7—C6	108.26 (10)	C15—N2—H2C	124.2
C8—C7—C6	102.82 (9)	C24—N3—C22	110.72 (11)
C9—C8—C5	118.03 (11)	C24—N3—C25	124.37 (13)
C9—C8—C7	113.84 (10)	C22—N3—C25	124.84 (12)
C5—C8—C7	105.66 (10)	C9—O1—C10	115.98 (15)
C9—C8—H8	106.2	N3—C25—H25A	109.5
C5—C8—H8	106.2	N3—C25—H25B	109.5
C7—C8—H8	106.2	H25A—C25—H25B	109.5
O2—C9—O1	123.47 (14)	N3—C25—H25C	109.5
O2—C9—C8	123.39 (15)	H25A—C25—H25C	109.5
O1—C9—C8	113.14 (12)	H25B—C25—H25C	109.5
C16—C11—C12	118.79 (15)	O1—C10—H10A	109.5
C16—C11—H11	120.6	O1—C10—H10B	109.5
C12—C11—H11	120.6	H10A—C10—H10B	109.5
C13—C12—C11	120.57 (16)	O1—C10—H10C	109.5
C13—C12—H12	119.7	H10A—C10—H10C	109.5
C11—C12—H12	119.7	H10B—C10—H10C	109.5
C12—C13—C14	121.43 (15)		
			
N1—C1—C2—C3	−54.22 (17)	C7—C6—C17—N2	−111.06 (11)
C1—C2—C3—C4	53.92 (19)	C23—C18—C19—C20	0.7 (2)
C2—C3—C4—C5	−55.34 (18)	C18—C19—C20—C21	0.0 (3)
C3—C4—C5—N1	58.10 (15)	C19—C20—C21—C22	−0.2 (2)
C3—C4—C5—C8	170.81 (12)	C20—C21—C22—C23	−0.3 (2)
N1—C6—C7—C23	147.66 (10)	C20—C21—C22—N3	176.32 (14)
C16—C6—C7—C23	−86.48 (13)	C19—C18—C23—C22	−1.2 (2)
C17—C6—C7—C23	26.31 (14)	C19—C18—C23—C7	179.05 (14)
N1—C6—C7—C24	−100.46 (11)	C21—C22—C23—C18	1.0 (2)
C16—C6—C7—C24	25.40 (14)	N3—C22—C23—C18	−176.14 (12)
C17—C6—C7—C24	138.19 (10)	C21—C22—C23—C7	−179.20 (12)
N1—C6—C7—C8	16.20 (11)	N3—C22—C23—C7	3.66 (15)
C16—C6—C7—C8	142.06 (11)	C24—C7—C23—C18	170.03 (14)
C17—C6—C7—C8	−105.16 (11)	C8—C7—C23—C18	48.5 (2)
N1—C5—C8—C9	−163.80 (11)	C6—C7—C23—C18	−73.96 (18)
C4—C5—C8—C9	78.22 (16)	C24—C7—C23—C22	−9.74 (13)
N1—C5—C8—C7	−35.14 (12)	C8—C7—C23—C22	−131.27 (12)
C4—C5—C8—C7	−153.12 (11)	C6—C7—C23—C22	106.27 (12)
C23—C7—C8—C9	15.14 (16)	C23—C7—C24—O4	−167.64 (13)
C24—C7—C8—C9	−101.89 (13)	C8—C7—C24—O4	−39.52 (17)
C6—C7—C8—C9	142.87 (11)	C6—C7—C24—O4	72.24 (16)
C23—C7—C8—C5	−115.95 (12)	C23—C7—C24—N3	12.89 (13)
C24—C7—C8—C5	127.02 (11)	C8—C7—C24—N3	141.01 (11)
C6—C7—C8—C5	11.78 (12)	C6—C7—C24—N3	−107.23 (11)
C5—C8—C9—O2	−163.28 (14)	C16—C6—N1—C1	66.27 (14)
C7—C8—C9—O2	72.00 (19)	C17—C6—N1—C1	−50.20 (15)
C5—C8—C9—O1	15.76 (18)	C7—C6—N1—C1	−167.55 (10)
C7—C8—C9—O1	−108.95 (14)	C16—C6—N1—C5	−166.61 (10)
C16—C11—C12—C13	1.4 (2)	C17—C6—N1—C5	76.93 (12)
C11—C12—C13—C14	1.0 (3)	C7—C6—N1—C5	−40.43 (11)
C12—C13—C14—C15	−1.8 (2)	C2—C1—N1—C6	−177.87 (11)
C13—C14—C15—C16	0.2 (2)	C2—C1—N1—C5	58.12 (15)
C13—C14—C15—N2	176.36 (14)	C4—C5—N1—C6	170.13 (10)
C12—C11—C16—C15	−2.9 (2)	C8—C5—N1—C6	48.25 (12)
C12—C11—C16—C6	178.76 (14)	C4—C5—N1—C1	−60.98 (14)
C14—C15—C16—C11	2.1 (2)	C8—C5—N1—C1	177.14 (10)
N2—C15—C16—C11	−174.62 (12)	O3—C17—N2—C15	169.09 (14)
C14—C15—C16—C6	−179.17 (12)	C6—C17—N2—C15	−9.49 (14)
N2—C15—C16—C6	4.08 (14)	C14—C15—N2—C17	−172.88 (13)
N1—C6—C16—C11	45.73 (19)	C16—C15—N2—C17	3.64 (15)
C17—C6—C16—C11	169.56 (14)	O4—C24—N3—C22	168.88 (13)
C7—C6—C16—C11	−73.29 (18)	C7—C24—N3—C22	−11.64 (14)
N1—C6—C16—C15	−132.75 (11)	O4—C24—N3—C25	−8.2 (2)
C17—C6—C16—C15	−8.92 (13)	C7—C24—N3—C25	171.31 (12)
C7—C6—C16—C15	108.23 (12)	C21—C22—N3—C24	−171.74 (14)
N1—C6—C17—O3	−43.25 (18)	C23—C22—N3—C24	5.19 (15)
C16—C6—C17—O3	−167.55 (14)	C21—C22—N3—C25	5.3 (2)
C7—C6—C17—O3	70.37 (16)	C23—C22—N3—C25	−177.78 (13)
N1—C6—C17—N2	135.32 (11)	O2—C9—O1—C10	1.1 (3)
C16—C6—C17—N2	11.02 (13)	C8—C9—O1—C10	−177.99 (17)

### Hydrogen-bond geometry (Å, º)

Cg6 is the centroid of the C18–C23 ring.

**Table:**

*D*—H···*A*	*D*—H	H···*A*	*D*···*A*	*D*—H···*A*
N2—H2*C*···O4^i^	0.86	2.21	2.9639 (15)	146
C2—H2*A*···O3^ii^	0.97	2.48	3.348 (2)	150
C25—H25*C*···*Cg*6^iii^	0.96	2.81	3.5617 (19)	135

Symmetry codes: (i) *x*−1/2, −*y*+1/2, *z*−1/2; (ii) *x*+1/2, −*y*+1/2, *z*+1/2; (iii) −*x*, −*y*+1, −*z*+1.
